# Problem-, resources- and goals-oriented (PRoGO) approach in psychiatry: illustrated by the case of Ludwig II of Bavaria

**DOI:** 10.1007/s00406-025-02192-9

**Published:** 2026-01-21

**Authors:** Stefan Leucht, John M. Davis, Kerem Böge, Amr ElDeeb, Wolfgang Strube, Hans Förstl

**Affiliations:** 1 Department of Psychiatry and Psychotherapy, Technical University of Munich, TUM School of Medicine and Health, Klinikum rechts der Isar, Munich, Germany; 2 German Center for Mental Health (DZPG), partner site Munich/Augsburg, Munich, Germany; 3 Psychiatric Institute, University of Illinois at Chicago, Chicago, USA; 4 Department of Psychiatry and Neuroscience, Charité – Universitätsmedizin Berlin, Berlin, Germany; 5 Department of Psychology, Medical University Brandenburg, Neuruppin, Germany; 6 German Center of Mental Health (DZPG), partner site Berlin/Potsdam, Berlin, Germany; 7 Kbo-Inn-Salzach-Klinikum Gabersee, Wasserburg am Inn, Germany; 8 Department of Psychiatry, Psychosomatics and Psychotherapy, University of Augsburg, Augsburg, Germany; 9 German Center for Mental Health (DZPG), partner site Munich/Augsburg, Augsburg, Germany; 10 Institut für Geschichte und Ethik der Medizin , Technical Universisty of Munich, TUM School of Medicine and Health, Munich, Germany

## Abstract

Mental health issues are dimensional in nature, yet both ICD-11 and DSM-5 are categorical classifications, leaving many patients not fitting into them. Using Bavaria’s “fairy-tale” king Ludwig II—whose diagnosis remains debated—as an example, we illustrate how the Problem-, Resources-, and Goal-Oriented (PRoGO) approach can lead to a better characterisation of patients and guide personalized treatment planning.

## Introduction

Several of the castles built by King Ludwig II of Bavaria (1845–1886) are now recognized as UNESCO World Heritage sites. Beyond his architectural legacy, he became the patron of Richard Wagner, at the time a notoriously debased political exile, heavily indebted, and on the brink of suicide. Without Ludwig’s backing, many of Wagner’s works might never have been created. Ludwig II died at the age of 40. The bodies of Ludwig and Bernhard von Gudden, a prominent psychiatrist at the time, were found dead in Lake Starnberg. The circumstances of his death remain shrouded in mystery. Ludwig had a number of "*mental issues*", but to this day, there is no consensus about his *real* diagnosis. Over the decades, many psychiatrists have attempted to identify his condition, but could not reach any agreement [1, 2].

In this editorial, we explain why diagnosing Ludwig II is an almost impossible task. Psychiatric terminology and diagnostic criteria have changed fundamentally over time. Furthermore, Ludwig exhibited multiple psychiatric symptoms, though most were not clear enough to allow for a definitive diagnosis. His case is a striking example of the two continua with which psychiatry struggles more than any other medical speciality.

The first may be referred to as the “vertical continuum”—the spectrum from health to severe illness. For example, most people experience periods of sadness, but it is difficult to determine when these states cross the line of clinically relevant depression. In the absence of objective biomarkers, we rely on subjective criteria such as personal distress and functional impairment to define the boundary between normal experience and disorder.

The second may be termed the “horizontal continuum”, referring to the overlap of symptom dimensions across different disorders. Individuals with schizophrenia, for instance, do not only exhibit positive, negative, cognitive, and motor symptoms; they also frequently experience affective symptoms, anxiety, and obsessive–compulsive symptoms.

Together, these two continua—combined with the lack of reliable biomarkers—make psychiatric diagnosis and treatment development inherently challenging. They also leave psychiatry particularly vulnerable to criticism, including extreme positions such as Michel Foucault’s claim that psychiatric illness is merely a social construct.

In this editorial, we use Ludwig II as a compelling example that prominently illustrates exactly these issues. Moreover, we want to show how the recently proposed *Problem-, Resources- and Goal Oriented* PRoGO approach [3, 4] would be more appropriate for diagnosing persons with mental problems than the current categorical systems ICD-11 and DSM-5.

## Brief biography

Ludwig II was the eldest of two sons of King Maximilian II and Queen Marie of Bavaria. His birth was protracted, and he suffered from meningitis as a small child. There was a family history of mental illness—an aunt and his younger brother Otto had a severe psychotic disorder.

In line with the educational norms of the time, Ludwig and his brother were raised in an emotionally distant manner rather than one of affection. Their parents and educators placed great importance on discipline and self-restraint, aiming to harden their sons for their future roles.

In early adulthood Ludwig developed a passion for music. When his father died unexpectedly, the then 18-year-old Ludwig was thrust onto the throne largely unprepared, and soon faced significant political challenges, including Prussia’s war with France. After the formation of the German Empire under hawkish Prussian leadership, Ludwig—who had aspired to rule like Louis XIV as an absolutist monarch—found himself a nominal king, sandwiched between a Prussian emperor managed by chancellor Bismarck, and the houses of representatives within his constitutional Bavarian monarchy.

One of the most painful episodes of his reign was the early public backlash over his generous support of composer Richard Wagner. The Bavarian ministers and citizens strongly opposed this patronage, ultimately forcing the king to dismiss him.

Ludwig never married. He once became engaged to Duchess Sophie Charlotte in Bavaria—the sister of Empress Elisabeth of Austria (“Sisi”)—but called off the marriage. There are good reasons to assume that the catholic king Ludwig struggled with his same-sex orientation, complicating his social contacts. Over time, he withdrew from public life and from his capital, Munich, spending his time in the countryside to devote himself almost entirely to his castle-building projects. He soon lost the charm and beauty of the tall, dark, handsome 18-year-old ascendant, became obese, lost most of his teeth, and suffered from chronic headaches .

Ultimately, the Bavarian government resolved to depose him due to the enormous debts he had incurred through his architectural projects. A panel of four leading psychiatrists, headed by Professor Bernhard von Gudden, diagnosed him with “paranoia” (“Verrücktheit” in German) —a concept that significantly overlaps with what is now called schizophrenia. Notably, they had not examined him in person, as it was evident he would have refused.

Ludwig was taken from Neuschwanstein Castle to his manor house Berg at Lake Starnberg. The next day, Whitsunday, June 13th 1886, Ludwig and von Gudden did not return from a walk in the park. Hours later, their bodies were found in the shallow water. Was it suicide? A failed escape attempt? Or were they both murdered? To this day, speculation and myth surround the final hours of the “fairy-tale king.”

## Diagnoses of Ludwig II

Over the past 150 years, numerous psychiatric diagnoses have been proposed for Ludwig II. One key reason for this diagnostic variability is the evolution of psychiatric terminology and classification systems over time. Von Zerssen attempted to map the diagnoses of 21 authors onto the ICD-10 classification system, yielding a total of 38 distinct diagnoses [1]. These ranged from neurodegenerative conditions such as early frontotemporal dementia (2), to a variety of schizophrenia spectrum disorders, as well as substance use disorders, social phobia, insomnia, and various personality disorders. Notably, 10 of the 21 authors diagnosed Ludwig with schizophrenia, a conclusion that appears inconsistent with the clinical features he exhibited.

## Outline of the problem-, resources and goal oriented (PRoGO)

We argue that the difficulty to assign a clear diagnosis to him is actually typical for everyday clinical practice. There are patients who fit well to the prototypes described by ICD and DSM. But many patients do not fit in. This problem is due to the dimensional nature of psychiatric disturbances. They are increasingly understood as network disorders rather than being the result of isolated, localized pathological lesions. This understanding explains the vertical and horizontal continua described above, as well as the high prevalence of psychiatric comorbidities, which are the rule rather than the exception [3].

Therefore, mental disorders align perfectly with the Problem-, Resources-, and Goals-Oriented approach (PRoGO) [4], an adaptation to psychiatry of Laurence Weed’s Problem-Oriented Medical Record (POMR) system [5].

First, this model emphasizes a comprehensive characterisation of the patient. This goes beyond a regular psychiatric and medical history, a description of psychopathological signs and symptoms and ruling out organic causes. It ideally encompasses cognition, IQ, personality, an assessment of the social situation, patients’ values and what brings meaning to a patient’s life. Moreover, traditional psychiatric care is often too deficit oriented. In contrast, resource orientation may offer greater support in fostering recovery.

In the PRoGO framework, the various clinical and contextual elements are conceptualised as “problems”. PRoGO does not attempt to force-fit patients into diagnostic categories that are not carved in nature but are ultimately decided by consensus. While ICD-11 and DSM-5 classifications remain essential for communication and administrative purposes, they should not be the central focus of psychiatric care [3].

Problems are then prioritized in collaboration with the patient, not only because shared decision-making is a patient right, but also because patients frequently hold values and goals that differ from those of clinicians, so that involving them may improve adherence.

## Application of the problem-, resources and goal oriented (PRoGO) to Ludwig II

### Characterisation of the king

**Fig. 1:**
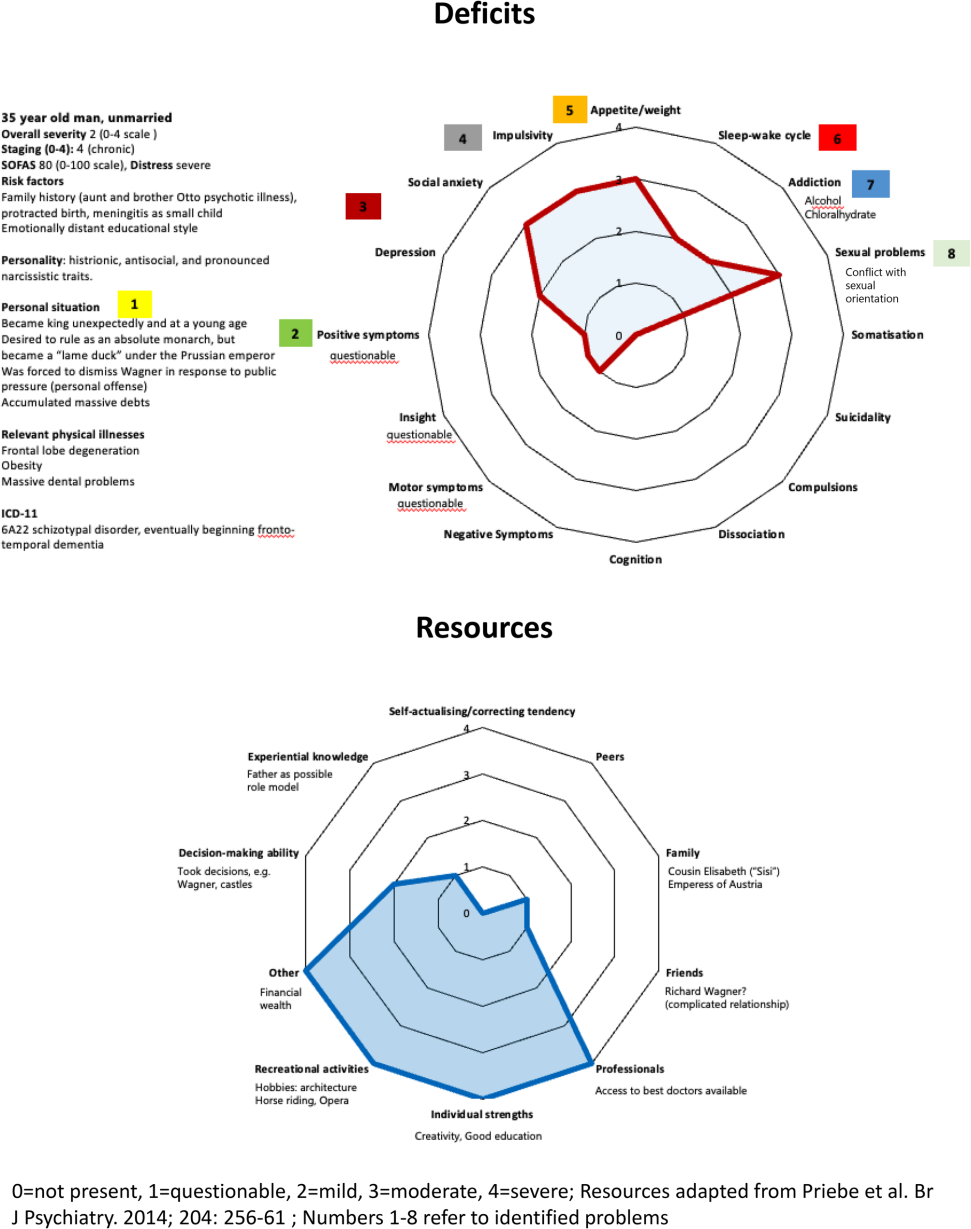
Characterisation of Ludwig II according to the PRoGO approach

The family history of severe psychotic illness, the protracted birth and his meningitis are well-established risk factors. The educational style at court contributed little to the development of social skills and emotional stability (Fig. 1, top). His personality exhibited increasingly histrionic, antisocial (e.g., physically assaulting his servants, contemplating bank robbery to resolve financial debts), and distinct narcissistic traits. Ludwig with his head in the clouds lost touch with the ground, first felt overwhelmed and soon became annoyed by the responsibilities of kingship. At times, he wanted his brother Otto to take his place. Early frustration (Wagner's forced dismissal, becoming a vassal of Prussia, etc.) and increasing social isolation have certainly aggravated his fragile personality's proclivity towards disastrous social dysfunction over time.

A central diagnostic question concerns the presence of positive psychotic symptoms. Accounts of the king isolating himself, speaking and laughing when alone, have been interpreted by some as signs of hallucinations—though this interpretation remains debatable [1]. Motor symptoms are questionable. Similarly, behaviours such as staging an imagined banquet in the snow with figures like Madame Pompadour, or his fantasies of descent from Louis XIV, are possibly better understood as overvalued ideas rather than true delusions. He probably retained the insight that he had constructed his dream world himself (Fig. 1, top).

It can be assumed that Ludwig was depressed. He showed signs of social anxiety, avoiding public appearances.

Ludwig’s impulsivity (aggressive outbursts and binge-eating, he became very obese) has been attributed to a possible frontal lobe dysfunction [2]. He had a disturbed sleep–wake cycle, famous are his nocturnal outings through the countryside in a horse-drawn sleigh. He took chloral-hydrate to induce sleep and opiates to manage headaches. He consumed alcohol, but he rarely drank to the point of completely losing control. It can be assumed that he experienced inner conflict regarding his homophilia (Fig. 1, top).

Within the ICD-11 or DSM-5 frameworks, a diagnosis of schizotypal disorder may best capture Ludwig’s subsyndromal psychotic state, accompanied by multiple psychiatric comorbidities [2]. This diagnosis is arguable, but not essential in the PRoGO framework anyhow.

### Characterisation of the king’s resources

The bottom of Fig. 1 presents the king’s resources. He was wealthy and would have had access to the best medical care at the time. He had an exceptional, if incomplete, education, and demonstrated remarkable creativity and energy in building his dream castles, forcing his architects and engineers to combine backwards-looking or outlandish designs with the latest technical achievements. Moreover, he was passionate about music.

### Prioritized problem list and treatment plans

Today, the following goal-oriented problem list and treatment plans could be derived. In practice, the king’s priorities would also be considered.

1. Challenges related to his role as king—a position for which he was insufficiently prepared.
  → Ideally, he would have had an advisor (like Bismarck) whose guidance he could accept, despite his narcissistic personality traits. Relying on such an advisor, he could spend more time on his true interests, architecture and music (resources).
2. Positive symptoms remain questionable.
  → a low dose of amisulpride (50 mg/day) and CBT for psychosis (CBT-p).
3. Anxiety/depression.
  → At 50 mg/day, amisulpride also exhibits antidepressant properties and is unlikely to exacerbate obesity.
4. Impulsivity (overeating, aggressive outbursts).
  → First, a diagnostic workup of potential frontal lobe dysfunction in light of a history of infantile meningitis or the possibility of beginning frontotemporal dementia would be needed.
5. Obesity.
  → Metformin, Exercise, Nutrition Counselling.
6. Flipped sleep–wake cycle.
  → Diagnostic workup, eventually CBT for Insomnia (CBT-I).
7. Alcohol, sleep- and pain medication use.
  → Psychoeducation, dental care, and eventually motivational interviewing.
8. Conflict with sexual orientation.
  → Cognitive behavioural therapy (CBT).

The king’s resource enormous wealth should be used to provide him with the best possible treatment.

### Computerised tools and follow-up using the SOAP scheme

L. Weed had also suggested using computerised tools to support diagnosis and treatment—an idea that with the advent of artificial intelligence is becoming a reality. Each problem should be followed up using the SOAP scheme (Subjective, Objective, Assessment, Plan) [5].

We have used Ludwig II as a case example for PRoGO. However, we argue that, due to the dimensional nature of mental disorders—and the resulting vertical and horizontal continua described above—such situations are the rule rather than the exception in psychiatry. While DSM and ICD classifications are essential for communication, epidemiological tracking, education, and billing, clinical practice should focus primarily on symptoms conceptualised as problems rather than consensus-based diagnostic categories [3, 4]. What’s the point of arguing about the name of the illness—what really matters is what you do about it.

## Data Availability

Not Applicable
